# An app for supporting older people receiving home care – usage, aspects of health and health literacy: a quasi-experimental study

**DOI:** 10.1186/s12911-020-01246-3

**Published:** 2020-09-15

**Authors:** Carina Göransson, Yvonne Wengström, Maria Hälleberg-Nyman, Ann Langius-Eklöf, Kristina Ziegert, Karin Blomberg

**Affiliations:** 1 Faculty of Medicine and Health, School of Health Sciences, Örebro University, 701 82 Örebro, Sweden; 2 School of Health and Welfare, Halmstad University, 302 18 Halmstad, Sweden; 3 Theme Cancer, Karolinska University Hospital, 141 86 Stockholm, Sweden; 4 Department of Neurobiology, Care Sciences and Society, Division of Nursing, Karolinska, Institutet, 171 77 Stockholm, Sweden

**Keywords:** Alert, App, Health, Health concerns, Home care, mHealth, Older people, Self-report, Usage

## Abstract

**Background:**

During the last decade, there has been an increase in studies describing use of mHealth, using smartphones with apps, in the healthcare system by a variety of populations. Despite this, few interventions including apps are targeting older people receiving home care. Developing mobile technology to its full potential of being interactive in real time remains a challenge. The current study is part of a larger project for identifying and managing health concerns via an app by using real-time data. The aim of the study was to describe older people’s usage of an app and to evaluate the impact of usage on aspects of health and health literacy over time.

**Methods:**

A quasi-experimental design was employed. Seventeen older people self-reported health concerns via Interaktor twice a week for 3-months and answered questionnaires at baseline, the end of the intervention and at a 6–month follow-up. Logged data on app usage and data on Sense of Coherence, Health Index, Nutrition Form for the Elderly, Geriatric Depression Scale-20, Swedish Communicative and Critical Health Literacy and Swedish Functional Health Literacy were collected and analysed using descriptive and non-parametric inferential statistics.

**Results:**

The median usage of the app as intended was 96%. Pain was one of the most reported health concerns and was also the health concern that triggered an alert (*n* = 33). The older people’s communicative and critical health literacy improved significantly over time. Regarding the scores of Sense of Coherence, Health Index, Nutritional Form for the Elderly, Geriatric Depression Scale-20 and Swedish Functional Health Literacy scale, there were no significant differences over time.

**Conclusions:**

The high app usage showed that an app may be a suitable tool for some older people living alone and receiving home care. The results indicate that the usage of Interaktor can support older people by significantly improving their communicative and critical health literacy. Aspects of health were not shown to be affected by the usage of the app. Further research with larger sample is needed for evaluation the effect on health literacy, and which aspects of health of importance to support by an app.

## Background

Older people have an increasing interest in using the internet to seek health-related information [1]. Even so, it has been concluded that they are less prone to seek health-related information on the internet than younger people, and when older people have several health problems the information seeking decreases [2, 3]. Understanding health-related information requires a certain level of health literacy, which is defined by the World Health Organization as the ability to “*gain access to, understand and use information in ways which promote and maintain good health*” [4]. It has been proposed that poor health literacy in the oldest old people can be a barrier both to seeking health information and to adopting mHealth [5].

MHealth has been described as the use of mobile technology to transfer health data in healthcare services as well as to seek and receive health information in order to improve health outcomes [6]. The use of mobile technology, such as computers, smartphones and tablets with integrated apps for collecting data and monitoring different conditions, is growing in the healthcare system [7]. Studies have focused on self-reporting and monitoring health problems related to specific chronic conditions such as diabetes or chronic heart failure [8, 9], and have shown significantly improved clinical outcomes, for example a decrease in HbA1c levels and blood pressure [10]. Even in those studies that focus on people’s self-reporting of health problems in apps, interactive components with healthcare professionals are rare [10]. To alleviate health problems an automatic feedback in the apps can be included, for instance in the form of self-care advice to the users [8].

In Sweden, an increasing proportion of older people with health problems are cared for in their own homes [11]. This puts demands on the healthcare system to develop innovative ways of coordinating care as well as to improve health literacy for older people so that they can utilize mHealth [12]. Studies including older people receiving home care and using mHealth interventions to alleviate health problems are limited [13]. Therefore, it is of importance to evaluate, and support, the use of mHealth to identify and enable early detection of health problems in older people.

To meet this need, our research group developed an interactive app, Interaktor, based on the theoretical framework of participation and person-centered care [14–16]. Patient participation is described as the person’s involvement in health and care, and having knowledge, as well as interaction with healthcare professionals [14]. Furthermore, person-centred participation is based on the person’s values and preferences, knowledge building and perceived taking control of their care [15]. Person-centered care also includes the person’s perspective of the care and the interaction as a partnership between the person receiving care and the healthcare professionals [16]. These concepts all emphasize the person’s values and knowledge, as well as strategies for taking an active role in their own health, and to perform activities for their health and self-care [14–16].

The app aims to support self-management of health concerns and has been developed for different populations [17–19]. The platform for Interaktor includes: (1) a component for assessment of the occurrence, frequency and distress level of health concerns; (2) connection to a monitoring web interface for healthcare professionals and logged data storage on a secure server; (3) a risk assessment model that sends alerts via short message service (SMS) to nurses; (4) continuous access to evidence-based self-care advice and links to relevant websites; and (5) graphs to view the history of reporting. The first version developed for older people receiving home care has been shown to be acceptable and user-friendly [20].

The aim of the study was to describe older people’s usage of an app and to evaluate the impact of the usage on aspects of health and health literacy over time.

## Methods

### Design

The design of this study was guided by the framework of The Medical Research Council’s for developing and evaluating complex interventions [21]. The framework’s three steps are: (1) define and understand the problem and the context; (2) develop the intervention; and (3) develop and optimize the evaluation. This quasi-experimental study is a part of the development and optimization phase that focuses on the outcomes of the intervention.

### Participants

**Fig. 1:**
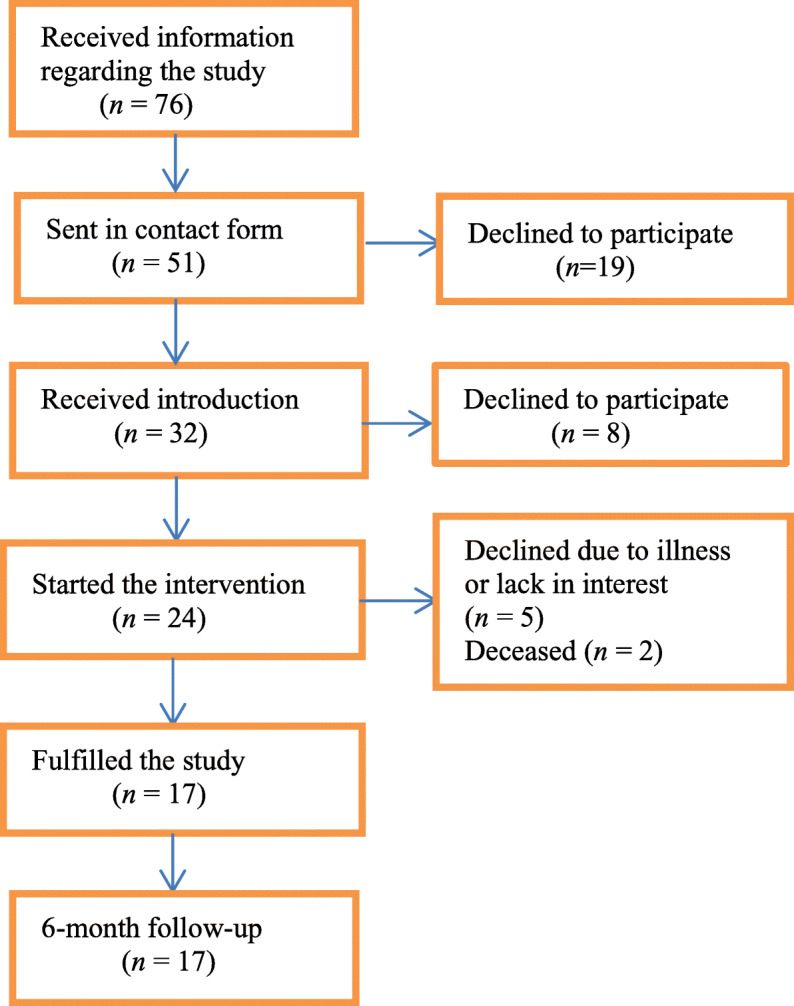
Flow chart over older persons participating in the intervention

The study was conducted in two municipalities in southwestern Sweden, one in rural area (site A) and one in an urban area (site B). The municipalities in Sweden are responsible by the laws set forth by the Health and Medical Services Act and the Social Services Act to offer home care services. These services are performed by different professions such as registered nurses working as homecare nurses [22, 23]. The homecare nurses identified eligible older people meeting the inclusion criteria: ≥65 years old, living in their own home, receiving home care, and having no cognitive impairment. In total 76 older people were identified and informed about the study by their homecare nurse, of those 76 older people a few were informed by a researcher during a meeting in one of the municipality’s centres for older people. Fifty-one submitted contact information, and were contacted by researcher and 19 chose at that point not to participate. The remaining 32 older people received information regarding the study and an introduction to the app and smartphone or tablet. The final sample consisted of 24 older people who used either a tablet (at site A, *n* = 15) or a smartphone (at site B, *n* = 9). Five older people dropped out, and two participants deceased, which left 17 participants who completed the study (Fig. 1). A more detailed description of the recruitment process for the participants has been published elsewhere [24, 25].

### The Interaktor app for older people receiving home care

The contents with different functions in the interactive app has been described in detail elsewhere [24]. In short, the 13 included health concerns were based on a literature review and interviews with older people and healthcare professionals [26]. The health concerns were assessed using standardized questions that included occurrence, frequency and distress level of the particular health concern [27, 28], which were programmed to trigger alerts based on algorithms. The algorithms were based on the older people’s self-reports of frequency and/or distress levels of the health concerns [24]. There were two kinds of alerts, yellow and red, depending on the severity of the health concerns and were sent instantly and automatically to the homecare nurses’ mobile phones. The homecare nurses contacted the older people in regard to their alerts and afterwards entered standardized notes into the system about the action taken, for example “contacted the patient”, “home visit” or “contact with physician”. The app includes access to self-care advice targeting older people and with links for further reading [29, 30]. Finally, the app allowed the older people to view the history of their reported health concerns in graphs over time. A more detailed description of the development of the contents in the Interaktor has been published elsewhere [24, 25].

### Procedure

The procedure of the intervention has been described in detail elsewhere [25]. The older people were provided with a smartphone or tablet on which Interaktor had been installed. They also received a code to log into Interaktor, as well as an identification number. They received written information from the researcher including screenshots of how to use the different functions in the app. The older people were instructed to submit a self-report twice a week on specific weekdays during the 3-month intervention, in total 26 times, and more often if needed. This was considered appropriate based on a previous study [20]. The homecare nurses were able to view the reported health concerns and alerts on the web interface.

### Data collection

#### Logged data

Data extracted from the database of logged data included: (1) the total number of participants’ self-reports; (2) the number of self-reports per health concern; (3) the occurrence, frequency and distress levels of health concerns; (4) the alerts generated; and (5) homecare nurses’ notes of actions based on generated alerts from the web interface.

#### Questionnaires

The older people received paper questionnaires at baseline, at the end of the intervention and at the 6-month follow-up. At baseline, the participants were given the questionnaires before the introduction of the smartphone/tablet and Interaktor. Some participants received support from the researcher who posed the questions verbally and filled out the questionnaires for them. After the intervention, the older people received the questionnaires when the smartphone/tablet was collected. At the 6-month follow-up the older people received the questionnaires by post and returned it in a prepaid envelope.

#### Aspects of health

The Sense of Coherence (SOC) scale measures how people cope, and also their general view of life, in dimensions of meaningfulness, manageability and comprehensibility [31]. It includes 13 items and respondents indicate agreement or disagreement on a 7-point scale, with two anchoring responses tailored to the content of each item. Five of the items are negatively stated and reverse-scored when calculating the total score. The total score range is 13–91; and a higher total score indicates better perceived health in general. The SOC scale has been psychometrically tested and has been found to be reliable and valid in different settings and in a variety of populations [31, 32]. The Health Index (HI) is a generic instrument for perceived general health [33]. It includes nine items and four response alternatives ranging from “very poor” to “very good”. The total score is minimum 9 and maximum 36. A higher score indicates better perceived health. The HI has shown to be reliable and valid both in hospital settings and in a population sample [33, 34]. The Nutritional Form for the Elderly (NUFFE) was used for screening the risk of undernutrition in older people [35]. This instrument comprises 15 items related to a person’s nutritional situation and has three response alternatives. The score ranges from 0 to 30. A score of 0–5 indicates that there is no risk of undernutrition, while a score of 6–12 indicates a moderate risk, and a score of ≥13 indicates a high risk of undernutrition. The NUFFE has shown reliable properties and has been validated in older people and rehabilitation settings [36]. The Geriatric Depression Scale (GDS)-20 is a screening tool for depression in older people [37]. It contains 20 dichotomous items (yes/no). A total score of > 5 indicates suspected depression. The GDS-20 has been described as reliable and valid in older people and for use in primary care centres [37].

#### Health literacy

The Swedish Functional Health Literacy (S-FHL) scale measures ability to read and understand health information [38]. It comprises five items with four response alternatives ranging from “never” to “often” [39]. The Swedish Communicative & Critical Health Literacy (S-C & C HL) scale is used to assess the skill to extract information and apply it [40]. It includes five items with five response alternatives, from “totally disagree” to “totally agree”. In both scales the score is calculated by collapsing the response alternatives into three: 1, 100 and 1000. The total score was calculated for each person and scores were categorized into three groups: sufficient < 100, problematic 100–1000, and inadequate > 1000 [41]. The S-FHL and S-C & C HL scales have been found to be reliable and valid [38, 40].

### Data analysis

Logged data were analysed with descriptive statistics. The older people’s usage of Interaktor for health reporting was calculated as the number of days an older person submitted a report, divided by the number of days that person was intended to submit a report, and presented as a percentage. The logged data of the self-reported health concerns were organized by frequency, where 1 = almost never; 2 = sometimes; 3 = often; and 4 = almost always, and also by how distressing the health concern was, where 1 = not at all; 2 = a little; 3 = pretty much; and 4 = very much.

In the analysis of the questionnaires, non-parametric tests were used [42]. To analyse differences over time the Friedman test for ordinal-level data, and Cochran’s Q test for nominal data were used. Differences between the three assessment points (baseline, end of the intervention, and 6-month follow-up) were analysed using the Wilcoxon signed-rank test for ordinal-level and McNemar’s test for nominal data [42]. All analyses were performed using IBM SPSS version 24.0. *P-*values (two-tailed) < 0.05 were considered statistically significant.

## Results

The mean age was 86 years (range 70–101), and eleven women and six men were included. Nearly all participants lived on their own. Fourteen participants had adult children who did not live with them. The most common medical diagnosis was cardiovascular diseases followed by musculoskeletal disease (Table 1).

**Table 1:** Participants’ sociodemographic and medical characteristics

Variables	(*N* = 17)
Age, yrs., mean (*SD*)	86 (6.5)
Female, *n* (%)	11 (64.7)
***Marital status, n*** **(%)**	
Married/living with partner	1 (5.8)
Living alone	16 (94.1)
***Education, n*** **(%)**	
Junior compulsory	11 (64.7)
Senior high school	1 (5.8)
College/university	4 (23.5)
Other education	1 (5.8)
***Medical diagnosis, n*** **(%)**	
Cardiovascular	15 (88.2)
Musculoskeletal	9 (52.9)
Respiratory	4 (23.5)

*SD* standard deviation

### The usage of the app

The logged data showed that the median usage of Interaktor was 96% (range 3–100%). For the group using a smartphone, the median was 83% (range 62–100%) and for tablet users, it was 100% (range 3–100%). The total number of self-reports submitted by the older people (*n* = 17) during the intervention was 383, and these 383 self-reports contained altogether 1253 health concerns. The most common self-reported health concerns were difficulties performing personal daily activities and difficulties performing social activities outside the home, followed by pain, fatigue, insomnia/sleeping problems, dizziness, worry, sadness, diarrhoea, constipation, loss of appetite, difficulty eating, and fever (Table 2). The frequency and distress levels varied for the included health concerns. All four levels of frequency and distress were reported (for the distribution of answers, see Table 2).

**Table 2:** Participants’ (*N* = 17) self-reported health concerns, by occurrence, frequency and distress level

Health concerns	Occurrence	Frequency		Distress	
	*n* (%)	Median (Q_1_-Q_3_)	Range	Median (Q_1_-Q_3_)	Range
Difficulties performing personal daily activities (*n* = 17)	262 (20.9)	3 (2–3)	1–4	2 (2–3)	1–4
Difficulties performing social activities outside the home (*n* = 17)	252 (20.1)	3 (2–3)	1–4	2 (2–3)	1–4
Pain (*n* = 13)	221 (17.6)	3 (3–4)	1–4	3 (2–3)	1–4
Fatigue (*n* = 14)	190 (15.1)	3 (2–4)	1–4	2.5 (2–3)	1–4
Insomnia/sleeping difficulties (*n* = 11)	87 (6.9)	3 (2–3)	1–4	2 (2–3)	1–4
Dizziness (*n* = 8)	71 (5.6)	2 (2–3)	1–4	2 (2–3)	1–4
Worry (*n* = 9)	50 (3.9)	2 (2–3)	1–4	2 (2)	1–4
Sadness (*n* = 11)	49 (3.9)	2 (2)	1–4	2 (2)	1–4
Diarrhoea (*n* = 7)	34 (2.7)	2 (2–3)	1–4	2 (2)	1–4
Constipation (*n* = 4)	14 (1.1)	2 (2)	1–4	2 (2)	1–4
Loss of appetite (*n* = 6)	12 (0.9)	3 (2–4)	1–4	2 (2)	1–4
Difficulties eating (*n* = 5)	6 (0.4)	1 (1–2)	1–4	1.5 (1–2)	1–4
Fever (*n* = 4)	5 (0.4)	2 (1–2)	1–4	2 (1–2)	1–4
**Total**	**1253**				

Frequency: 1 = almost never; 2 = sometimes; 3 = often; and 4 = almost always. Distress levels: 1 = not at all; 2 = a little; 3 = pretty much; 4 = very much. Q_1_ = first quartile; Q_3_ = third quartile

Of the self-reported health concerns, 79 generated alerts to the homecare nurses, 74 were yellow (less severe) and five were red alerts (severe). The most common self-reported health concern that triggered alerts was pain (yellow, *n* = 33) (Table 3). The total alerts (yellow and red) were generated by 14 of the participants, with a median of five alerts (minimum one alert, maximum 16) during the time of using Interaktor.

**Table 3:** Distribution and category of alerts linked to self-reports by the participants (*N =* 14) during the intervention

Health concerns	Yellow alerts *n* (%)	Red alerts *n* (%)
Pain (*n* = 9)	33 (44.5)	
Dizziness (*n* = 4)	20 (27.0)	3 (60.0)
Diarrhoea (*n* = 3)	14 (18.9)	2 (40.0)
Fever, 3 days (*n* = 2)	2 (2.7)	
Sadness, 2 days (*n* = 1)	1 (1.3)	
Difficulties eating (*n* = 1)	1 (1.3)	
Fever (*n* = 1)	1 (1.3)	
Constipation (*n* = 1)	1 (1.3)	
Diarrhoea and fever (*n* = 1)	1 (1.3)	
**Total alerts**	**74**	**5**

Yellow alert: the homecare nurse contacted the participant within 24 h

Red alert: the homecare nurse contacted the participant on the same day

Of the 74 yellow alerts, 35 led to the homecare nurses taking actions and making notes (Table 4). The actions taken by the homecare nurses when they received an alert included home visits (*n* = 10) and contact with the patient (*n* = 6). When the first alert came in, the homecare nurses made the most notes, but when subsequent alerts came in for the same health concern, they either noted “no action” or stopped making notes. In 39 alerts, no comments were noted. In 19 of these, the alerts were sent by the same two older people regarding the same health concern, pain.

**Table 4:** Notes made by the homecare nurses when receiving alerts from the participants (*N* = 14)

Notes	Yellow alerts, *n*	Red alerts, *n*
Home visit	10	1
No action	10	
Contacted the patient	6	2
Telephone contact	4	
Booked visit with the patient	1	
Tried to reach the patient – no answer	1	
Patient had contact with the physiotherapist	1	
Information received – no action taken	1	
Is sorted out; nothing new	1	
No action; alert probably due to participant pressing the wrong key		1
No notes made	39	1
**Total**	**74**	**5**

Yellow alert: the homecare nurse contacted the participant within 24 h

Red alert: the homecare nurse contacted the participant on the same day

### Aspects of health and health literacy

The statistical analysis did not show any significant changes over time for the included instruments measuring aspects of health (Table 5). The older people’s SOC scores showed a small, but not significant, increase at the end of the intervention compared with baseline and at 6-month follow-up. The participants’ general health was unchanged from baseline to the end of the intervention and the 6-month follow-up. The older people had a moderate risk of undernutrition, from baseline to 6-month follow-up. The results indicated no suspected depression at any of the three measurement points. The older people’s functional health literacy (Swedish FHL Scale) did not differ significantly between baseline, the end of the intervention and the 6-month follow-up. Their communicative and critical health literacy, indicated by the Swedish C & C HL Scale, had improved by the 6-month follow-up compared with both baseline (*p* = 0.02) and the end of the intervention (*p* = 0.02) (Table 6).

**Table 5:** Comparison of the median scores for the included instruments at baseline, at the end of the intervention and at 6-month follow-up (*N* = 17)

Instruments	Range		Median (Q_**1**_-Q_**3**_)					
		Baseline	End of intervention	6-month follow-up	***P***-value^a^	***P***-value^b^	***P***-value^c^	***P***-value^d^
**SOC scale**	13–91	63 (59–70)^γ^	66 (59.7–74.5)^β^	64 (59–73)^π^	0.08’	0.75’	0.07’	0.11^▫^
**HI**	9–36	24 (23–28.7)^β^	24.5 (21.2–28)^β^	25 (22–28)^π^	0.50’	0.06’	0.17’	0.33^▫^
**NUFFE**	0–30	6 (4–9.5)^γ^	8 (4.5–9.5)^γ^	7 (5–9)^π^	0.30’	0.52’	0.13’	0.65^▫^
**GDS**	0–20	3 (2.2–5.5)^ε^	5 (2–9)^π^	5 (4–8)^π^	0.12^α^	0.21^α^	1.00^α^	0.15^∞^

*GDS* Geriatric Depression Scale, *HI* Health Index, *NUFFE* Nutritional Form for the Elderly, *SOC* Sense of Coherence. Q_1_ = first quartile; Q_3_ = third quartile

^a^Baseline vs end of the intervention; ^b^Baseline vs 6-month follow-up; ^c^End of the intervention vs 6-month follow-up; ^d^Comparison over time the three assessments points

^‘^Wilcoxon signed rank test; ^▫^Friedman’s test; ^∞^Cochran’s Q-test; ^α^McNemar’s test

^γ^*n* = 17; ^β^*n* = 16; ^π^*n* = 15; ^ε^*n* = 12

**Table 6:** The participants’ (*N* = 17) levels of health literacy at baseline, at the end of the intervention and at 6-month follow-up

Health literacy levels		Time points					
	Baseline *n*	End of intervention *n*	6-month follow-up *n*	***P***-value^a^	***P***-value^b^	***P***-value^c^	***P***-value^d^
**Swedish FHL scale,** ***n***				1.00’	0.73’	0.48’	0.77^▫^
Sufficient	5	3	4				
Problematic	9	13	7				
Inadequate	3	1	4				
Missing	0	0	2				
**Swedish C & C HL scale,** ***n***				0.25’	0.02’	0.02’	0.01^▫^
Sufficient	2	3	8				
Problematic	9	9	3				
Inadequate	5	3	3				
Missing	1	2	3				

*FHL* Functional Health Literacy, *C & C HL* Communicative and Critical Health Literacy

^a^Baseline vs end of the intervention; ^b^Baseline vs 6-month follow-up; ^c^End of the intervention vs 6-month follow-up; ^d^Comparison between all three assessment points

^‘^Wilcoxon signed rank test; ▫Friedman’s test

## Discussion

The Interaktor usage seems to have improved the older people’s health literacy, concerning their ability to extract health information and apply it, as seen at the 6-month follow-up. This result is interesting, as health literacy is known to decline with old age [5], and low health literacy has been described elsewhere as an obstacle to older people seeking for health information and using mHealth [3, 43]. It has also been reported that old age and having several medical conditions can have a negative impact on using technology to seek health information [2]. Using an app can confirm older people’s prior knowledge and increase their self-confidence in self-care [24]. The improvement in health literacy for the older people in the current study may be due to the fact that the included information and links to websites were specifically targeted to an older population [30]. When information is adapted to older people’s situation and interests, the likelihood is that it will be used [44]. Another explanation for the improved health literacy could be the instant access to health information in a new way that Interaktor provided. This may have stimulated the older people after completing the intervention to seek more health information and apply it. It has been reported that older people’s health literacy can be stimulated by the use of mHealth [5].

The older people’s aspects of health did not change during the 3 months that they used Interaktor. The short intervention time may have affected the possibility to improve aspects of health. It has been shown that specific health outcomes can be enhanced by self-reporting via smartphone; however, the intervention time varies between studies from 2 months to 1 year [9, 10], indicating that deciding what time period is the most suitable for accurate evaluation of health outcomes can be challenging. Another reason for the unchanged aspects of health in the current study may be the older people’s advanced age, in comparison to participants in the aforementioned studies, most of whom were < 70 years old [9, 10]. Studies targeting older people with chronic conditions using mHealth have shown that the impact on health can vary, demonstrating a range of impact from improvement to no effect [5, 8]. However, to self-report on a regular basis can be a reminder of illness [45]. For the older people in the current study, this does not seem to have been the case, as the aspects of health remained unaffected. Therefore, it is important to further evaluate the Interaktor app in home care, in terms of which aspects of health are most important and can be supported by using an app.

The most health concerns self-reported by the older people were difficulties performing personal daily activities, difficulties performing social activities outside home and pain. The reason for the high frequencies of difficulties performing activities may be the high median age (86 years) of the older people included here, as it is well known that physical ability in daily life becomes more of an issue with advanced age [46]. Compared with the past, today more older people are living in their own home rather than in nursing homes, and receiving help with activities of daily living (ADLs) [47]. By giving these people the opportunity to self-report their health concerns via an app, the homecare nurses can detect any change in ADL function at an early stage and take relevant action. It is of importance to self-report difficulities performing personal activities and social activities with others, as physical ability and health are known to be interrelated [48].

Pain was likewise a highly self-reported health concern among the older people participating in the present study. This is in line with results from another study describing pain as a common health concern in older people [49], although it has also been stated that the prevalence of pain can vary among older people [50]. Another reason for the high self-reporting frequencies could be that the app gave the older people a new opportunity to communicate pain, as also described in our previous study [24]. This is important as it is concluded that insufficient assessment by healthcare professionals and communication regarding older people’s pain can occur [50].

Pain was the health concern which triggered most alerts. The reason for this could be that the level for triggering alerts for pain was set too low. Once an older person’s pain was well known to the homecare nurses and had been repeatedly reported at the same frequency and distress level, the nurses did not always write notes about actions taken. This may be so because the nurses were already aware of the level of the pain problem; or else it may have been due to their heavy workload, as described previously [24].

The older people’s high app usage can be explained by our previous results, which describe the Interaktor as easy to use as well as including health concerns that are relevant to older people [24]. Studies have shown lower frequency of app usage for people between 50 and 79 years old with different specific chronic conditions and in a variety of settings [51, 52]. Other studies in different populations have shown high usage of Interaktor [17–19]. The high usage in the current study may also reflect older people’s interest in using new technology, which is in line with other studies pointing out that using new technology can prompt learning in older people [44]. Another reason for the high usage may be the trust the older people had in the homecare nurses when asked to participate in the study. This was described by the older people as making them feel modern and acknowledged as a valued person [24]. The homecare nurses may have played a role in inspiring the older people to use the app. It is stated that social influence impacts the use of new technology [53]. In the current study the majority of the older people lived on their own, and did not receive encouragement to use the app from a partner. Although the majority of the older people had adult children only a few mentioned the children’s support when using the app [24]. In further studies it would be of interest to include family members’ perspective and evaluate the impact of the older people’s usage.

### Strengths and limitations

The main limitation of this study was the small sample size. The homecare nurses identified more older people than were included as eligible for the study. However, not all invited people agreed to participate. This may indicate that some older people may have lacked confidence in using this new technology [43]. The older people who participated in this research had fewer health problems and medical diagnoses compared with older people receiving home care in general [24], which may have affected the results in terms of fewer reported health concerns, alerts and unchanged aspects of health. Furthermore, healthier older people are more likely to participate in studies compared with non-healthy people [54].

One strength of the study is the context of home care, since interventions using mHealth targeting older people in home care are limited [13]. More intervention studies are needed as the use of smartphones and tablets by older people is increasing [3].

The results should be generalized with caution [55]; nevertheless, they indicate outcomes of importance for studies including larger samples. Larger studies performed in patients with prostate cancer during radiotherapy and in patients after surgery of pancreatic cancer have shown that the use of the app Interaktor could contribute to alleviate the health problems [18, 19]. However, it has been argued that to conduct studies with smaller samples within the field of mHealth is appropriate as the technology is developing rapidly [56]. To conduct small studies is also recommended in preparation for large-scale evaluation according to the framework for complex intervention [21]. Further studies with larger samples are needed to evaluate the impact of using Interaktor on aspects of older people’s health and health literacy before implementation in home care.

## Conclusions

This study indicates that an interactive app can be used as a tool for health concerns by some older people living alone and receiving home care. The older people’s communicative and critical health literacy was improved by their usage of the app; but there was no significant improvement in aspects of health. The high usage of the app by the older people indicates the importance of including the increasing older population in the continued development of mHealth to be better integrated in home care for reporting health concerns in real time. Further research including larger samples and a longer intervention time is needed for evaluation of the effect of using applications of older people’s health literacy, and which aspects of health are most important and can be supported by using an app.

## Data Availability

The dataset used and analysed during the current study is available from the corresponding author upon reasonable request.

## Abbreviations

app: application
GDS-20: Geriatric Depression Scale-20
HI: Health Index
mHealth: mobile health
NUFFE: Nutritional Form for the Elderly
S C & C HL: Swedish Communicative and Critical Health Literacy Scale
S FHL: Swedish Functional Health Literacy Scale
SMS: Short Message Service
SOC: Sense of Coherence
